# The tricot approach: an agile framework for decentralized on-farm testing supported by citizen science. A retrospective

**DOI:** 10.1007/s13593-023-00937-1

**Published:** 2024-01-25

**Authors:** Kauê de Sousa, Jacob van Etten, Rhys Manners, Erna Abidin, Rekiya O. Abdulmalik, Bello Abolore, Kwabena Acheremu, Stephen Angudubo, Amilcar Aguilar, Elizabeth Arnaud, Adventina Babu, Mirna Barrios, Grecia Benavente, Ousmane Boukar, Jill E. Cairns, Edward Carey, Happy Daudi, Maryam Dawud, Gospel Edughaen, James Ellison, Williams Esuma, Sanusi Gaya Mohammed, Jeske van de Gevel, Marvin Gomez, Joost van Heerwaarden, Paula Iragaba, Edith Kadege, Teshale M. Assefa, Sylvia Kalemera, Fadhili Salum Kasubiri, Robert Kawuki, Yosef Gebrehawaryat Kidane, Michael Kilango, Heneriko Kulembeka, Adofo Kwadwo, Brandon Madriz, Ester Masumba, Julius Mbiu, Thiago Mendes, Anna Müller, Mukani Moyo, Kiddo Mtunda, Tawanda Muzhingi, Dean Muungani, Emmanuel T. Mwenda, Ganga Rao V. P. R. Nadigatla, Ann Ritah Nanyonjo, Sognigbé N’Danikou, Athanase Nduwumuremyi, Jean Claude Nshimiyimana, Ephraim Nuwamanya, Hyacinthe Nyirahabimana, Martina Occelli, Olamide Olaosebikan, Patrick Obia Ongom, Berta Ortiz-Crespo, Richard Oteng-Fripong, Alfred Ozimati, Durodola Owoade, Carlos F. Quiros, Juan Carlos Rosas, Placide Rukundo, Pieter Rutsaert, Milindi Sibomana, Neeraj Sharma, Nestory Shida, Jonathan Steinke, Reuben Ssali, Jose Gabriel Suchini, Béla Teeken, Theophilus Kwabla Tengey, Hale Ann Tufan, Silver Tumwegamire, Elyse Tuyishime, Jacob Ulzen, Muhammad Lawan Umar, Samuel Onwuka, Tessy Ugo Madu, Rachel C. Voss, Mary Yeye, Mainassara Zaman-Allah

**Affiliations:** 1 Digital Inclusion, Bioversity International, Montpellier, France; 2 Department of Agricultural Sciences, Inland Norway University of Applied Sciences, Hamar, Norway; 3 International Institute of Tropical Agriculture (IITA), Kigali, Rwanda; 4 Reputed Agriculture 4 Development Stichting & Foundation, Kumasi, Ghana; 5 Department of Plant Science, Institute for Agricultural Research, Ahmadu Bello University, Zaria, 810211 Nigeria; 6 International Institute of Tropical Agriculture (IITA), Ibadan, Nigeria; 7 Savanna Agricultural Research Institute, Council for Scientific and Industrial Research, Tamale, Ghana; 8 National Crop Resources Research Institute, Kampala, Uganda; 9 Centro Agronómico Tropical de Investigación y Enseñanza, Managua, Nicaragua; 10 Tanzanian Agricultural Research Institute, Arusha, Tanzania; 11 International Maize and Wheat Improvement Center (CIMMYT), Harare, Zimbabwe; 12 Lake Chad Research Institute, Lagos, Nigeria; 13 One Acre Fund, Kigali, Rwanda; 14 Centre for Dryland Agriculture, Bayero University, Kano, Nigeria; 15 Digital Creativity Lab, University of York, York, UK; 16 Fundación para la Investigación Participativa con Agricultores de Honduras (FIPAH), La Ceiba, Atlántida Honduras; 17 Department of Plant Sciences, Wageningen University and Research, Wageningen, The Netherlands; 18 School of Life Sciences and Bioengineering, The Nelson Mandela African Institution of Science and Technology, Arusha, Tanzania; 19 Crops for Nutrition and Health, International Center for Tropical Agriculture (CIAT), Arusha, Tanzania; 20 Biodiversity for Food and Agriculture, Bioversity International, Addis Ababa, Ethiopia; 21 Council for Scientific and Industrial Research-Crops Research Institute, Kumasi, Ghana; 22 MrBot Software Solutions, Cartago, Costa Rica; 23 International Potato Center (CIP), Nairobi, Kenya; 24 Department of Food, Bioprocessing and Nutrition Science, Raleigh, NC USA; 25 Dryland Legumes and Cereals, International Maize and Wheat Improvement Center (CIMMYT), Nairobi, Kenya; 26 World Vegetable Center (ARVDC), Arusha, Tanzania; 27 Rwanda Agriculture and Animal Resources Development Board (RAB), Huye, Rwanda; 28 International Potato Center (CIP), Kigali, Rwanda; 29 College of Agriculture and Life Sciences, Cornell University, Ithaca, NY USA; 30 Genética y Fitomejoramiento, Escuela Agrícola Panamericana Zamorano, Tegucigalpa, Honduras; 31 Sustainable Agrifood Systems, International Maize and Wheat Improvement Center (CIMMYT), Nairobi, Kenya; 32 Tuberosum Technologies Inc., Broderick, Saskatchewan Canada; 33 Humboldt University Berlin, Berlin, Germany; 34 International Potato Center (CIP), Kampala, Uganda; 35 Centro Agronómico Tropical de Investigación y Enseñanza, Esquipulas, Guatemala; 36 Forest and Horticultural Crops Research Center, University of Ghana, Accra, Ghana; 37 Institute for Agricultural Research (IAR), ABU, Zaria, Nigeria; 38 National Root Crops Research Institute, Umudike, Nigeria

**Keywords:** External validity, Participatory plant breeding, Socio-economic heterogeneity, Target product profile

## Abstract

Matching crop varieties to their target use context and user preferences is a challenge faced by many plant breeding programs serving smallholder agriculture. Numerous participatory approaches proposed by CGIAR and other research teams over the last four decades have attempted to capture farmers’ priorities/preferences and crop variety field performance in representative growing environments through experimental trials with higher external validity. Yet none have overcome the challenges of scalability, data validity and reliability, and difficulties in capturing socio-economic and environmental heterogeneity. Building on the strengths of these attempts, we developed a new data-generation approach, called *triadic comparison of technology options* (tricot). Tricot is a decentralized experimental approach supported by crowdsourced citizen science. In this article, we review the development, validation, and evolution of the tricot approach, through our own research results and reviewing the literature in which tricot approaches have been successfully applied. The first results indicated that tricot-aggregated farmer-led assessments contained information with adequate validity and that reliability could be achieved with a large sample. Costs were lower than current participatory approaches. Scaling the tricot approach into a large on-farm testing network successfully registered specific climatic effects of crop variety performance in representative growing environments. Tricot’s recent application in plant breeding networks in relation to decision-making has (i) advanced plant breeding lines recognizing socio-economic heterogeneity, and (ii) identified consumers’ preferences and market demands, generating alternative breeding design priorities. We review lessons learned from tricot applications that have enabled a large scaling effort, which should lead to stronger decision-making in crop improvement and increased use of improved varieties in smallholder agriculture.

## Introduction

To favor adoption of new crop varieties, crop breeding programs need to ensure these varieties are matched to targeted use contexts. This is particularly challenging in breeding for smallholder agriculture, as crop growing environments tend to be heterogeneous and crop product requirements are directly linked to their use for domestic consumption (Walker and Alwang 2015; Thiele et al. 2021). In the late 1970s and early 1980s, international agricultural researchers at CGIAR realized that they needed to engage future technology users earlier and more directly in the technology development process through participatory approaches. In a pioneering study, Rhoades and Booth (1982) involved farmers from the outset in a technology design process (focused on potato storage), which spurred local innovation processes and adoption of the resulting technologies. In this same period, breeding programs also started to use participatory methods (Ceccarelli and Grando 2019). Participatory plant breeding (PPB) can involve farmers from the beginning of the selection process, whereas participatory variety selection (PVS) involves farmers only in the selection of (near-)finished breeding products. Participatory trials were often based on conventional experimental designs implemented on farms or consisted in inviting farmers to the research station to observe trial entries (Sperling et al. 1993).

In spite of the known advantages of farmer participation, at present few breeding programs in CGIAR use a PPB approach (Ceccarelli and Grando 2019). In the past, CGIAR social scientists have expressed concern over the mismatch in methodological approaches applied by social scientists on the one hand and plant breeders and other natural scientists on the other (Bebbington and Carney 1990; Thiele et al. 2001; Cernea and Kassam 2005; German et al. 2010). Although CGIAR implemented a 14-year program that included PPB and social and gender inclusion (BiermayrI-Jenzano et al. 2011), its impact remained very limited because of the impossibility to breed for the many different user niches addressed as PPB case studies. This hampered the identification of cross-cutting preferences that could be cost-effectively addressed by an affordable number of breeding pipelines in which significant added value (genetic gain) can be created. At the same time, the limited adoption rate of new crop varieties has remained a reason for concern (Walker and Alwang 2015; Thiele et al. 2021).

Decentralizing crop genotype testing in farmers’ fields faced a range of challenges. Perceived limitations of participatory methods in breeding include concerns about scalability (Atlin et al. 2001), limitations in getting robust insights from the data (Coe 2002), and difficulties capturing socio-economic and environmental heterogeneity, which had so far been treated generally as a random nuisance factor (van Etten et al. 2023). Based on a detailed study, Misiko (2013) has documented how a participatory trial worked in practice. In his case study, farmer-led trials were difficult to manage, as farmers did not always fully appropriate the trials, had limited motivation to take good care of the trials, and had to deal with free riding. Participating farmers visited the trial only a few times, and based their judgment mainly on their final snapshot during the field day, rather than consistent observation throughout the crop season. Only when they planted the seeds on their own farms, farmers would discover the real performance of the new varieties, which often contrasted with their impressions during field day. As a result, variety adoption was low, even among participating farmers.

Decentralized, participatory on-farm testing was still rare in the early 2000s (Morris and Bellon 2004). A highlight in this period, causing renewed enthusiasm for participatory trials, was the introduction of mother and baby trials (MBT) by Snapp (2002). MBT was described as a decentralized approach that involved farmers as active participants testing the crops in their own farms to capture biological performance and farmers’ priorities, and improve external validity as a high client-oriented approach (Witcombe et al. 2006). In this approach farmers had the opportunity to select a group of varieties (up to three, plus one check) to test on-farm and contribute to the information generated in the mother trials (centralized researcher-managed trials, often placed on farms). The approach was recommended across CGIAR (Atlin et al. 2002). It has probably been the most commonly used decentralized participatory plant breeding approach in the last decade (De Haan et al. 2019). Despite these efforts, the majority of CGIAR breeding programs have not consistently used MBT or other participatory approaches in recent years. With few exceptions, participatory or on-farm trials are often limited in scope and run on an ad hoc basis. One other shortcoming is that farmers, especially in the Global South, are considered providers of feedback rather than equal partners in the variety development process. Farmers receive very little recognition for their contribution to crop breeding. This contrasts with, for example, how potato farmers in the Netherlands initiated breeding efforts and were part and parcel of it (Lammerts van Bueren et al. 2018).

In an attempt to develop a scalable and inclusive participatory variety selection approach, van Etten (2011) revisited these experiences highlighting their strengths, which are (i) farmer participation, (ii) client-oriented design, (iii) data collection in representative heterogeneous environments, and (iv) focus on external validity. Some weaknesses of the existing participatory approaches highlighted were (i) evaluations as snapshots; (ii) assessments with a rating approach, which can lead to bias; (iii) uncoordinated and duplicative efforts in trial design and data collection; and (iii) lack of comprehensive geographical coverage. Van Etten (2011) proposed to develop an alternative approach following experiences in crowdsourcing citizen science in ecology and environmental sciences, which successfully engage motivated volunteers in research (Cooper et al. 2014). Van Etten et al. (2016) describe the resulting approach, tricot, and the first experiences in its implementation. The approach tested four crops in three continents, in a total of 16,000 farmer-managed experimental plots. Twelve years later, tricot is in a scaling phase across the Global South having reached ~ 150,000 farmer- managed plots testing several crops covering cereals, legumes, vegetables, roots, and tuber crops.

In this paper, we describe the experiences gained with tricot over the last 12 years. The focus is on how the approach was developed and improved to align with the farmers, plant breeders, and social scientists needs by delivering robust data and timely insights for crop variety management. We examine five development phases of the tricot approach: (i) proof of concept; (ii) data-driven approach; (iii) mainstreaming in breeding programs; (iv) challenges of mainstreaming in breeding programs; and (v) informing product design. We conclude by discussing the use of tricot to understand gender and socio-economic heterogeneity, recent lessons from scaling across breeding programs, and innovation issues that need to be addressed in future research and implementation. The aim is to describe how tricot evolved and what is done with it, how it is being modified and used within public breeding initiatives in the endeavor to acquire better-informed, systematic, and more representative feedback from crop users, especially in an era where public breeding objectives and donors stress the need for social impact at scale and genetic gain within farmers’ fields.

## The tricot approach

In its initial phase, tricot was called “crowdsourced crop improvement,” but the name was changed to avoid calling participants “crowds,” which is not respectful of their role. Tricot is a more neutral name, which is short for *triadic comparison of technology options.* This refers to an incomplete block design with a block size of three technology options, which is characteristic of the approach. The idea of involving many participants as active citizen scientists during the whole crop cycle remained the key principle of tricot.

**Fig. 1:**
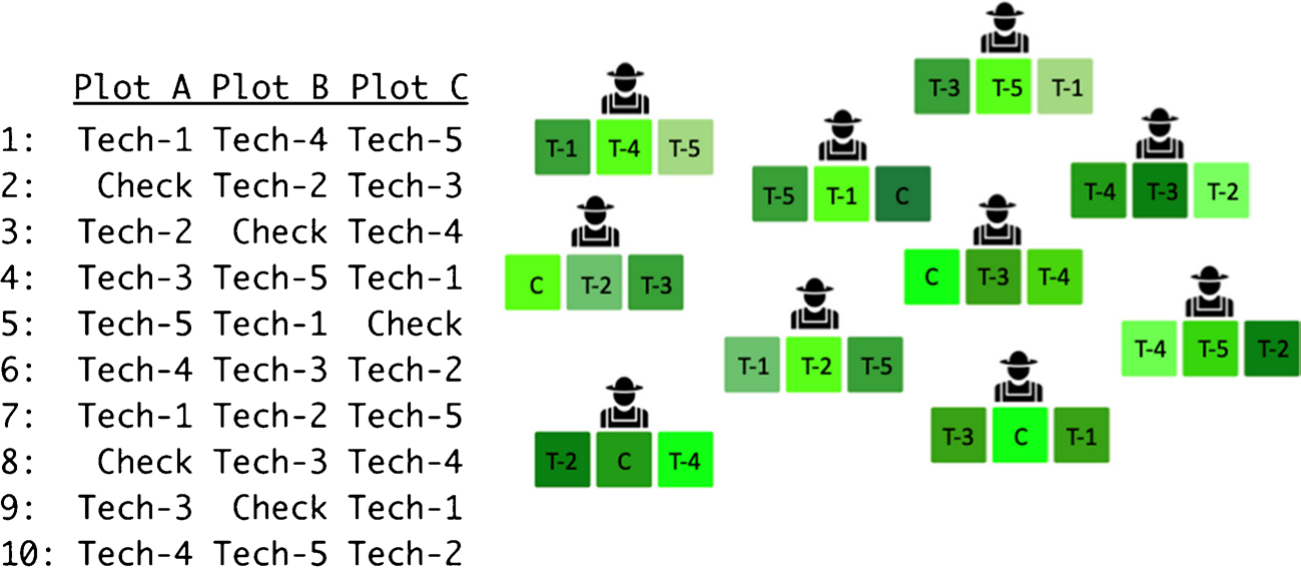
Illustration of an A-optimal incomplete block design implemented by tricot. Rows are the blocks and columns are the treatments. In this example five technologies were tested, named Tech-1 to Tech-5, with the addition of one check (control treatment). This is a simplification as more than one check is recommended in reality. Check is part of the design that follows the principles of clinical trials. Balanced A-optimal design ensures a cohesive experimental structure even when blocks are systematically lost. Packages (blocks) should be distributed to participants in ordinal sequence (from 1 to *n*).

As described by van Etten et al. (2016), the experimental principles of tricot build on existing on-farm testing and decentralization concepts, including those introduced by Snapp (2002) with MBT. Tricot combined different existing approaches to ensure a robust experimental design in farmer-led experiments (van Etten et al. 2016). The first element was the use of a balanced incomplete block design with a block size of three technology options (Fig. 1). The design ensures A-optimality, which means that trials are as robust as possible in connecting all technology options to each other in the set, even when blocks are lost due to self-attrition or external causes (Bailey and Cameron 2009). Another characteristic of the approach was masking the entry names to avoid farmer bias to the greatest degree possible. Farmers are informed about the names of the technology options that they evaluated by the end of the experiment.

The second element was the co-development of a digital platform to support the experimental design, data management, and reporting, the ClimMob digital platform (Quirós et al. 2023), a free on-line software available at https://climmob.net/. This platform supports trial managers with the trial design and provides a standardized database for efficient data utilization and open collaboration. This is unusual practice in crop experimentation and on-farm research, as data are often still stored locally or never digitized properly, and data are rarely combined across seasons or trials to extract insights (Brown et al. 2020; Valle et al. 2022).

**Fig. 2:**
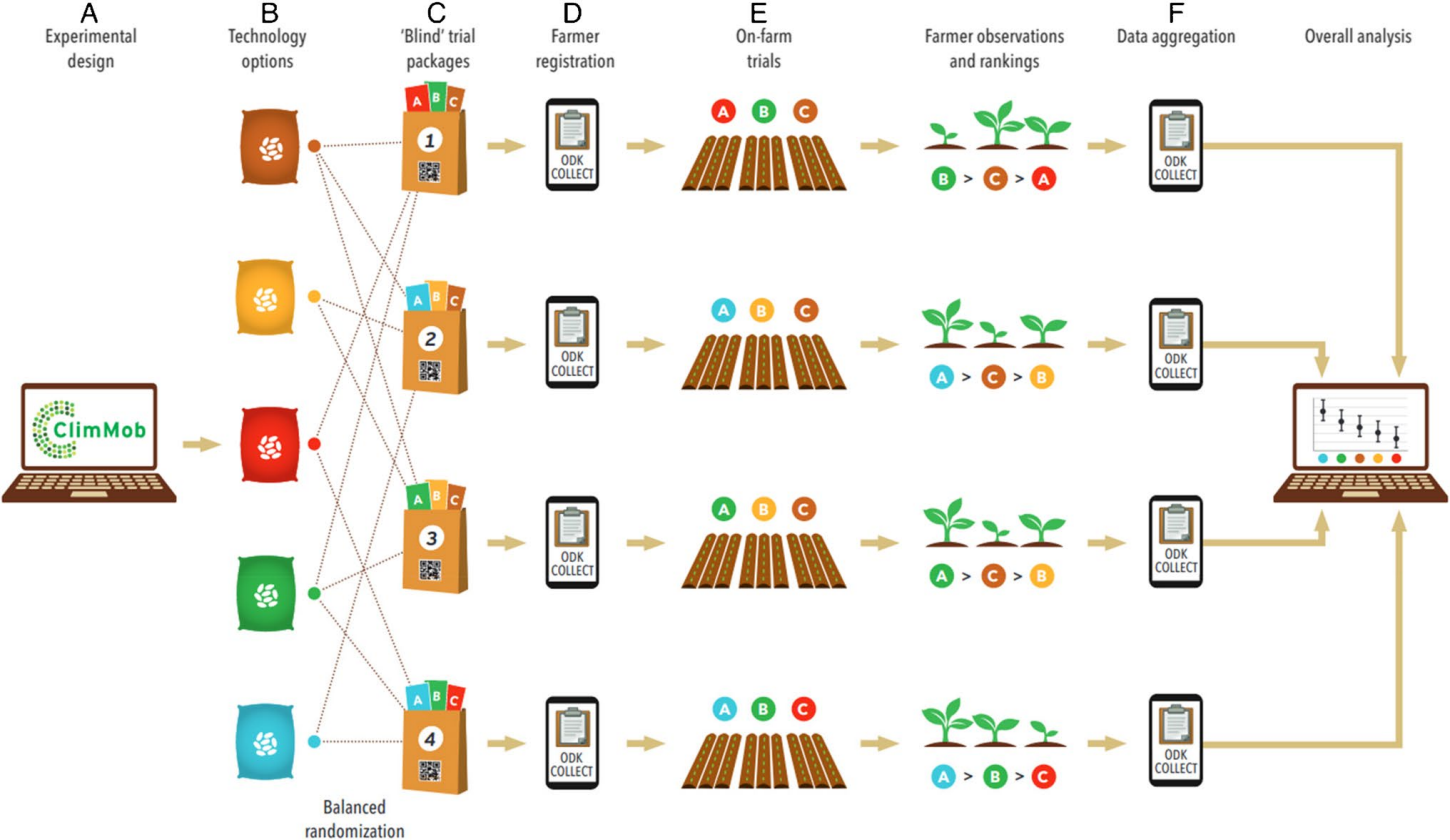
Overview of the tricot trial design and implementation. (A) Trials are designed on ClimMob (https://climmob.net/) following a trial protocol derived from the target product profile. (B) Technology options (varieties, breeding lines, etc.) are selected based on the aims of the experiment. (C) Sets of three technology options are randomly assigned to a trial package as incomplete blocks of three following an A-optimal design. (D) Field agents distribute the trial packages and register participants’ identifiable data (name, age, village, district, GPS) using Open Data Kit. (E) Participants establish the experiment in their own farm and assess a list of traits as per trial protocol (e.g., drought tolerance, yield) using “tricot rankings” by indicating the option with best performance (1st in the ranking) and the option with worst performance (3rd in the ranking) for the given trait. The 2nd place in the ranking is added to the option not mentioned as best or worst for the given trait. (F) Participants’ assessments are registered using Open Data Kit, sent to ClimMob, and aggregated for data analysis and production of automated reports. In consumer and market testing, step “(E)” is not considered and participants’ assessments are recorded at registration.

The principles of active farmer participation and robust experimental design remained unchanged since the first implementation of tricot trials and are at the core of the tricot approach as they are crucial in ensuring statistical explanatory power while still embracing the environmental diversity across many farmers’ fields. In Fig. 2 we describe these principles as steps in the implementation of tricot trials. The next steps, from farmer registration to overall analysis, were co-developed and improved in a close iterative processes with delivery teams and are described in the next sections of this paper. Step-by-step instructions on the implementation of on-farm trials with tricot are described in the tricot manual (van Etten et al. 2020).

## A proof of concept

From 2011 to 2015, van Etten et al. (2016) tested tricot as a proof of concept for a scalable on-farm testing approach in India, Ethiopia, and Central America (Nicaragua, Honduras, El Salvador, and Guatemala). The activities were integrated into regional and global initiatives. The work was part of the CGIAR Research Programme on Climate Change and Food Security (CCAFS). In India, the work was implemented within the CGIAR-India collaboration framework. The Ethiopian work was part of the Seeds for Needs Initiative (Fadda et al. 2020). In Central America, the work was implemented with the Mesoamerican Agro-environmental Program (Gutiérrez-Montes and Ramirez-Aguero 2015). Trials had different goals for each of the regions of implementation. In India, the goal was to increase crop varietal diversity by introducing new varieties of bread wheat (*Triticum aestivum* L.) and rice (*Oryza sativa* L.) into farmers’ fields (Gotor et al. 2021). In Ethiopia, the goal was to characterize the accessions of durum wheat (*Triticum durum* L.) registered in the Ethiopian national genebank and explore their potential for reviving this crop (Mancini et al. 2017; Kidane et al. 2017). In Central America, the goal was to increase the adoption of drought-tolerant common bean (*Phaseolus vulgaris* L.) varieties in dry areas affected by El Niño Southern Oscillation (ENSO).

**Fig. 3:**
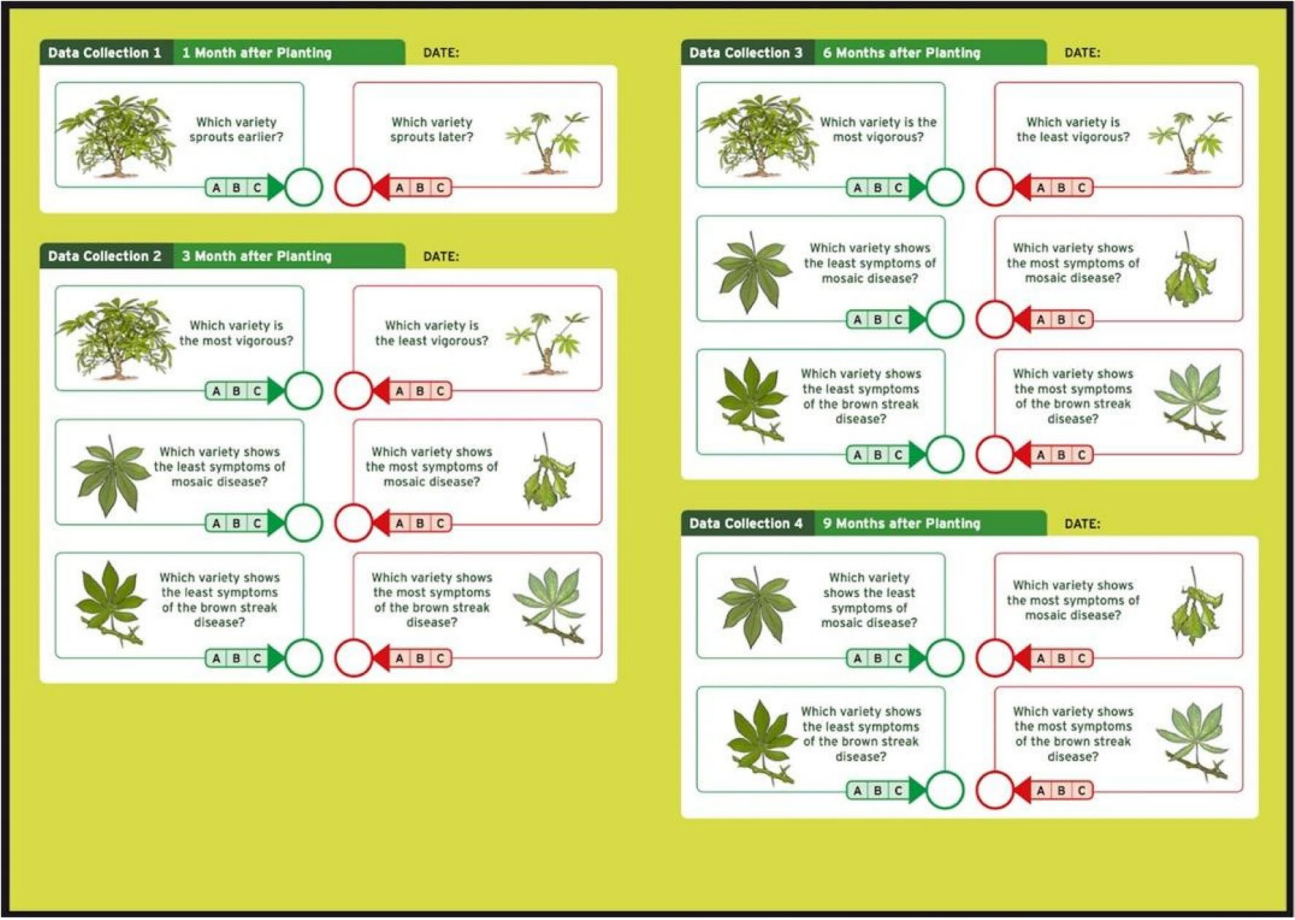
English translation of the data collection booklet for cassava (*Manihot esculenta* Crantz) trials in Rwanda. In each pair of illustrations, the one on the left represents the positive extreme (for example, “most vigorous”), while the one on the right represents the negative extreme of the same trait (“least vigorous”). The booklet is provided with clear illustrations to allow interpretation even by illiterate farmers, but also in the local language (Kinyarwanda) to improve engagement with the local partners. For each question on the card, a farmer writes the corresponding letters in the circles.

On-farm trials were implemented in collaboration with local partners who were supplied with technical and financial resources for the activities. A first round of capacity building focusing on the utilization of ClimMob and implementation of tricot was provided. This phase was based on four questions (i) What motivates farmers to participate in tricot? (ii) What is the accuracy of farmer-generated data in the agricultural trials? (iii) Is tricot cost efficient compared to existing participatory approaches? (iv) Can tricot improve the adoption of new crop varieties? Data collection materials were provided in the local language to enhance participant engagement and used clear illustrations to allow illiterate farmers to participate on equal terms (Fig. 3).

To answer the first question about farmers’ motivation, Beza et al. (2017) conducted semi-structured interviews with 426 farmers in the three regions of study. The authors found that key motivations were the opportunity to contribute to scientific research and to share information and interact with experts. Few farmers expected monetary recognition, but many emphasized seed innovation (keeping the seeds) in India, capacity building in Honduras, and agronomic advice in Ethiopia. Recently, farmers’ motivations were assessed in other countries. In a recent interaction in Uganda (2021−2022), farmers expected recognition of their work. We considered offering farmers co-authorship, similar to Mancini et al. (2017), as a symbolic recognition of their contribution. More open-ended interviews in Kenya, however, showed that farmers were especially interested in local social recognition and suggested that this could be realized with a t-shirt with a logo that represented the project or trial activity (van de Gevel 2022). One current incentive is allowing farmers to keep the harvest from the plots, even from breeding lines, as part of recognition (except for hybrid varieties, or when national regulations forbid it). In a recent review of variety adoption in Africa, Thiele et al. (2021) indicated that farmers keeping the seeds and sharing them through farmers’ seeds systems is an important driver of rapid variety adoption.

To address the second question about the accuracy of farmer-generated data, Steinke et al. (2017) compared the data from evaluations independently performed by farmers and breeders in Honduras. The resulting data indicated that the aggregated farmers’ assessments contained information with adequate validity and that low reliability should be compensated with a higher number of samples to generate meaningful results. These findings were in line with previous work in bird ecology, where researchers dealt with low reliability using a high volume of data and proper statistical methods (Cooper et al. 2014). In our experience, a tricot trial with around 12 entries (varieties, lines, etc.) should typically cover ~ 150 farms (with 37 readings per entry) to provide variety recommendations. This will give a “large” effect size (smaller is better), a Cohen’s* d* of 0.8 (Cohen 1988; Talsma 2018). This means that the trial has a 95% probability of identifying differences of 0.8 standard deviations. We think that generally a Cohen’s *d* of 0.8 is acceptable since trials are conducted over several seasons, as this metric will diminish over time if checks and most varieties are tested during more than one season. Recruiting a large number of farmers and producing enough seeds can be a challenge for the first round of tricot trials. For the first season, trial managers are advised to recruit only 50 farmers as a pilot exercise and focus on acquiring experience with the approach. For the subsequent seasons, scaling can be done in collaboration with local farmers’ organizations or extension services who generally possess a large network of farmers.

Thirdly, assessing the cost efficiency of the tricot versus existing participatory approaches was an important consideration for the adoption of tricot as a feasible experimental approach. We developed a systematic method for calculating the cost efficiency of tricot vs a benchmark state-of-art randomized complete block design (RCBD). We tested this approach in Rwanda during two seasons of tricot trials with maize (*Zea mays* L.) varieties, comparing costs incurred in tricot against costs incurred in RCBD. We established 155 tricot trials with 11 maize varieties, with a further 50 RCBD trials with 6 varieties. This means that there were 465 experimental units (3 × 155) under tricot and 300 under RCBD. To estimate the cost efficiency of each trial design, we calculated their relative statistical power and cost per experimental unit. As tricot follows an A-optimal incomplete block design with a block size of three, all other factors being equal, a balanced incomplete block design is less efficient than a RCBD per experimental unit. We captured the efficiency of an incomplete block, relative to a RCDB, using the following equation (Bailey and Cameron 2009):

1 $$E=t/(t-1)*(k-1)/k$$

where *t* is the number of treatments and *k* is the block size. This assumes that plot heterogeneity does not play a role. Plot heterogeneity is difficult to assess. The average mutual distances between varieties within blocks are smaller in tricot plots than in RCBD plots, so in principle this should favor tricot. Our calculation is conservative in this regard.

The formula implies that the relative efficiency of tricot for the use case study is 0.73. We take this into account by reducing the experimental units to “equivalent experimental units” (EEU). Each EEU is equivalent to 1 RCBD experimental unit in an RCBD with the same overall number of varieties. This means that tricot had 341 equivalent experimental units (Table 1).

**Table 1:** Comparative cost analysis (per experimental unit) of potato trials in tricot vs randomized complete block design (RCBD) in Rwanda.

Variable	Tricot	RCBD
Participants	155	50
Plots	155	50
Varieties	11	6
Experimental units	465	300
Equivalent experimental units (EEU)	341	300
Type of costs (USD)		
Seeds	283.04	522.72
Fertilizer	25.28	65.24
Material	390.58	106.33
Transport	263.25	404.35
Airtime	78.26	78.26
Labor	476.77	652.17
Total costs (USD)	1517.18	1829.07
Cost-effectiveness per EEU (USD)	4.45	6.10

This demonstrates that tricot costs 27% less than RCBD. Part of the cost reduction is due to the reduced seed requirement, as tricot subplots (experimental units) were smaller than the RCBD subplots. Even so, tricot plots were still considered to be of an adequate size to avoid strong edge or competition effects. The cost of tricot trials is, however, country specific, but experiences from other countries show the same trend when comparing tricot vs RCBD. In Central America, tricot has shown to be ~ 40% cheaper than RCBD, with higher costs in phone calls (to obtain farmers’ assessments) and seed packaging (Occelli et al. 2024).

Finally, Occelli et al. (2024) assessed the farmers’ likelihood in adopting new crop varieties after one or more rounds of tricot trials. For this, the authors conducted a study in the Trifinio transborder region between Guatemala, El Salvador, and Honduras, where they compared farmers’ adoption and benefits in tricot vs a benchmark group-based PVS. For this group of farmers, the authors obtained baseline data (in 2015) and endline data (in 2018), including a comprehensive household characterization (Hammond et al. 2017; van Etten et al. 2019b).

Adoption is not the immediate goal of on-farm testing (in contrast with demonstration plots), but measuring adoption provides a direct measure of how well the trials were able to produce locally relevant information about the suitability of new crop varieties. Occelli et al. (2024) defined as an “adopter” farmers who declared at the endline (three years after the experiment) to have planted and harvested a variety received by either participating in the PVS or the tricot approach within. This was a conservative approach as the literature considers that new varieties evaluated in on-farm experiments generally need up to 10 years for adoption and circulation within farmers’ seed systems (Thiele et al. 2021). In Occelli et al. (2024), out of the 19 evaluated varieties, 7 were not yet released to the market at the time of the endline evaluation in 2018, and 3 were released the following year (in 2019). In this case, the authors were capturing adoption levels in a relatively short window of time.

The results from the study revealed that farmers involved in PVS and tricot had comparable levels of variety adoption (Occelli et al. 2024). Out of 394 PVS participants, 74 (18%) affirmed to still harvest the variety delivered after the intervention; similar figures apply to tricot farmers, whose adoption rate was 20% after 3 years (146 adopters out of 739 participants). In absolute terms, adoption rates are in line with existing estimates of variety adoption (Walker and Alwang 2015). Since the tested varieties were mostly new cultivars, an average of 20% adoption rate is considered promising, but still not superior to the benchmark. This raised a concern on whether target product profiles (TPP)—the blueprint for the design of new varieties that indicates the traits and characteristics required in a new variety (Donovan et al. 2022)—in breeding programs are addressing farmers’ needs during the design progress. Or whether the delivery of a non-preferred variety in the tricot package affects farmers’ adoption decision. New studies on these issues are being developed. We discuss the first concern (design and TPP) in the next section of this paper.

## Data-driven approach

During the subsequent data-driven phase, van Etten et al. (2019a) tested the ability of tricot data to provide robust, actionable information on crop variety recommendations to heterogeneous groups across different agro-climatic zones. As an analytical approach, van Etten et al. (2016) proposed the Bradley-Terry model (Bradley and Terry 1952) combined with model-based recursive partitioning (Zeileis et al. 2008) to estimate the probability of one variety being selected over the others. This framework followed the Luce’s choice axiom, which was proposed within the context of behavioral sciences (Luce 1959). Bradley-Terry models provided robust insights and much more information than the usual response tables or principal component analysis adopted in participatory approaches in agriculture (Coe 2002; van Etten et al. 2016). However, the model presented limitations. Rank-breaking, converting rankings into pairwise comparisons, was needed to make the tricot data fit the Bradley-Terry model. This strategy has been used before (Dittrich et al. 2000) with other types of rankings, but it implies a loss of information, and underestimates *p* values and confidence intervals, a bias that cannot be corrected analytically (Zhang 2021). A permutational approach avoids this problem but requires additional computational effort.

Having the analytical tools implemented in R (R Core Team 2020) was a must for the team involved in the data analysis, as a way to ensure open data collaboration, reproducibility, and repurposing the data. With that in mind, the team collaborated with Turner et al. (2020) to develop an R language implementation of the Plackett-Luce model (Luce 1959; Plackett 1975). The Plackett-Luce model also follows Luce’s choice axiom but estimates the coefficients (*worth* parameters) using the complete ranking instead of pairwise comparisons. This makes it possible to compare items across the entire rank permutation.

With the former issues solved, the analysis of tricot data could be expanded using a high volume of data. In the first assessment, van Etten et al. (2019a) used data from 12,409 tricot plots from Nicaragua, India, and Ethiopia. The results showed that tricot data can register specific effects of climatic variation on variety performance and generate generalizable spatial predictions of seasonal crop variety performance. The authors applied the *worth* parameters from the Plackett-Luce model to perform risk assessment analysis and identify the variety with higher performance under uncertain climate scenarios. In the same study, the *worth* parameters were also used to estimate the probability of outperforming a check (Eskridge and Mumm 1992), an important breeding metric for the advancement or release of new crop varieties, a concept closely related to the *win rate*, the number of times, or locations where the new crop variety outperforms the check. Recently, de Sousa et al. (2023a) expanded this framework by combining quasi-variance estimates (Firth and De Menezes 2004) and Bayesian bootstrap (Rubin 1981) to perform risk assessment considering the confidence intervals in the variety performance.

**Fig. 4:**
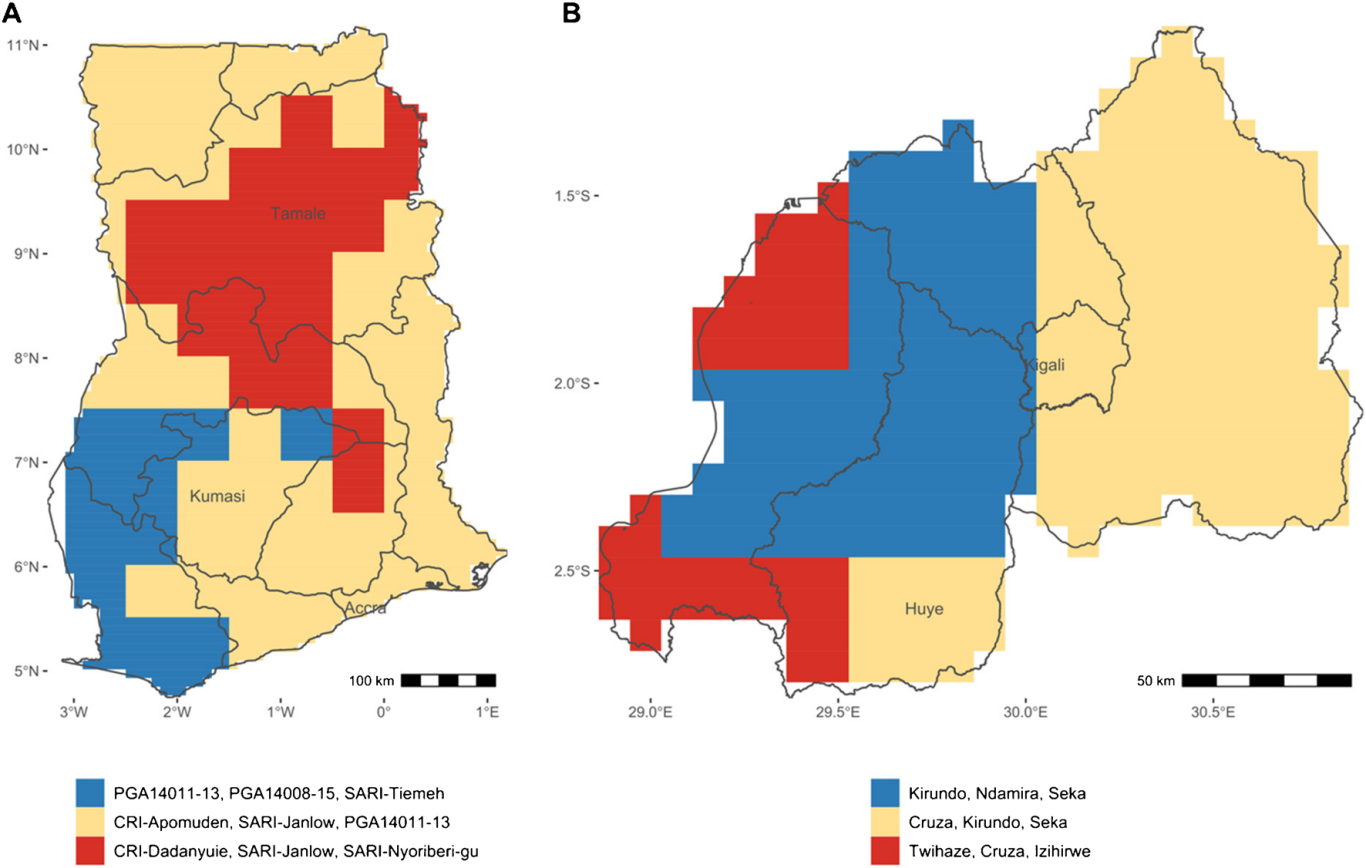
Crop variety recommendations from a simulated crop season based on predictions from Plackett-Luce models using climatic variables for the main crop season for **A** sweet potato in Ghana and **B** potato in Rwanda. Map categories show the top-three varieties for each area according to their *worth* estimates.

Two recent case studies on the potential of tricot data to provide variety recommendations with seasonal and geographical extrapolation can be provided. Firstly, in Ghana, 17 recently released or pre-release varieties of sweet potato (*Ipomoea batatas* (L.) Lam.) were evaluated across six regions (Fig. 4A). Trials were conducted in collaboration with government research and extension services and involved a total of 1268 farmers and 93 extension agents. Farmers ranked their varieties for agronomic and harvest traits and provided their overall preference. Rankings for overall performance were combined to produce a Plackett-Luce model on the basis of seasonal agroclimatic indices for the maximum duration of dry days (precipitation < 1 mm) and the maximum duration of summer days (temperature > 35 °C, Fig. 4A). SARI-Janlow, a newly released variety, showed high performance when exposed to long periods of consecutive dry days and high day temperature during the crop season, and should be in high demand by farmers. PGA14011-13, one of the advanced selections, performed very well in southern trials under intermediate periods of consecutive dry days, and may be put forward for release.

In the second case study, in Rwanda, 11 pre-release and released potato (*Solanum tuberosum* L.) varieties were tested across seven districts. Figure 4B outlines the results from aggregated data of 135 trials conducted in the first season. The Plackett-Luce model produced nodes based on seasonal agroclimatic indices for the daily temperature range (difference between day and night temperature) and the maximum duration of rainy days (precipitation > 1 mm), showing clear agronomic performance for certain varieties in the highland regions of Rwanda (red blocks), compared to lower areas in blue and yellow. Although trials were managed individually by different institutes in Rwanda, ClimMob can pool data from all sources to extract recommendations across larger and more diverse areas, enabling collaborative decision-making.

Data analysis has recently expanded into two directions. Firstly, a methodology to synthesize tricot data and deliver insights from independent trials (as illustrated for Rwanda above) has been described in detail by Brown et al. (2022), including a characterization of uncertainty of the modeling results. Secondly, de Sousa et al. (2021) analyzed tricot on-farm trial data with genomic relatedness data as a covariance matrix in a Bayesian framework, which increased the predictive power of the model in an important measure. This shows that it may be feasible and relevant to use genomic data to allow more diverse sets of materials to be tested by farmers. This provides prospects for breeding, as discussed in the next section.

Yildiz et al. (2020) have developed a new regression approach for ranking data based on the Plackett-Luce model, which was implemented in an update of the R package PlackettLuce (Turner et al. 2020). This model adds “item covariates” to the Plackett-Luce model. This makes it possible to model farmers’ and consumers’ rankings as a linear combination of different trait measurements or breeding values obtained from on-station trials or instrumental analysis. Recently, this framework has been applied by Olaosebikan et al. (2023) and Alamu et al. (2023) to assess drivers of consumers’ preferences of *gari* and *eba* (cassava sub-products) in Nigeria and Cameroon. Ongoing work uses biological measurements from on-station trials as item covariates to explain farmers’ variety preferences.

Data analysis has also been increasingly supported by implementing the existing code, which was generated to a large degree for the analyses developed by van Etten et al. (2019a), into R packages (de Sousa et al. 2020a, b, 2023a, b; Turner et al. 2020) and are used to produce the automated reports on ClimMob (de Sousa et al. 2022). Since 2020, more than 200 implementing partners made up of agricultural professionals (breeders, agronomists, social scientists) have benefitted from capacity building (virtual and in-person) on tricot data analysis. Lessons learned from this phase are being incorporated into a formal curriculum to improve capacity building on the independent design, implementation, and analysis of tricot data. Cassava breeding in Nigeria is also developing initiatives to integrate tricot data with BreedBase (https://breedbase.org/), the centralized database used by CGIAR and national partners for cassava, yam, and banana (Agbona et al. 2023). The process of standardizing crop traits within ClimMob through the utilization of the Crop Ontology (Shrestha et al. 2012) is an integral component of the effort to seamlessly integrate ClimMob with BreedBase. This integration facilitates the exchange of data stored in both systems, thereby paving the path for broader accessibility to tricot data and analytical insights for breeders.

## Mainstreaming in breeding programs: three case studies

Recently, tricot has been employed as an approach to inform breeding programs on the advancement of breeding lines and subsequent release. Within a breeding pipeline, early-generation screening is conducted on several hundred lines. The number of lines at each stage decreases significantly as only the selected lines advance to the next stage. To estimate the genetic value of a line, all lines must be tested at the same location. Given the large number of lines tested in early generations, on-farm testing has primarily been used at the last stage of breeding to validate the performance of new varieties within the target population environment (Gaffney et al. 2016). Tricot can facilitate early generation on-farm testing by employing genomic selection combined with sparse testing, providing new opportunities to predict the genetic value of haplotypes without testing each candidate line at all locations. This approach has been tested by de Sousa et al. (2021) using data from 1100 durum wheat on-farm trials in Ethiopia showing the ability of tricot to detect locally adapted genotypes with superior performance. The digital platform used within tricot allows fast data turnaround, a key requirement when implementing a genomic selection scheme within a demand-driven timeline. As breeding programs move to increase the number of seasons per year, the decision maker needs to be able to predict the value of haplotypes prior to the next stage.

The experience gained/proof of concept in the use of tricot to support breeding decisions can be illustrated with three case studies. First, CIMMYT’s maize breeding program, which has conducted extensive on-farm trials to deliver genetic gain to farmers and generate evidence of it. Collinson et al. (2022), provided the first evidence for a single-gene technology in maize that conferred a significant yield increase in low-yielding farm environments through an on-farm trial. However, they used an approach for on-farm testing that was not scalable. Until recently, on-farm testing in the maize program was conducted using a randomized complete block design with 20−30 hybrids replicated three times at each farm site. This approach not only proved to be quite complex given the number of hybrids to be evaluated but had also some implications with regard to data quality and relevance. To address those limitations, the tricot approach was adopted both in eastern and southern Africa during 2021 and 2022 for five maize target product profiles. This shift has translated into improving data relevance and quality through (i) significant network expansion testing (from less than 50 farms to more than 800 farms in 2022, Fig. 5); (ii) inclusion of farmers with very small fields because of a significant reduction of block size in tricot; (iii) ease of trial management, including harvesting, and reduction of errors during data collection; and (iv) reduction of uncoordinated spatial spread of the trials leading to less experimental noise.

**Fig. 5:**
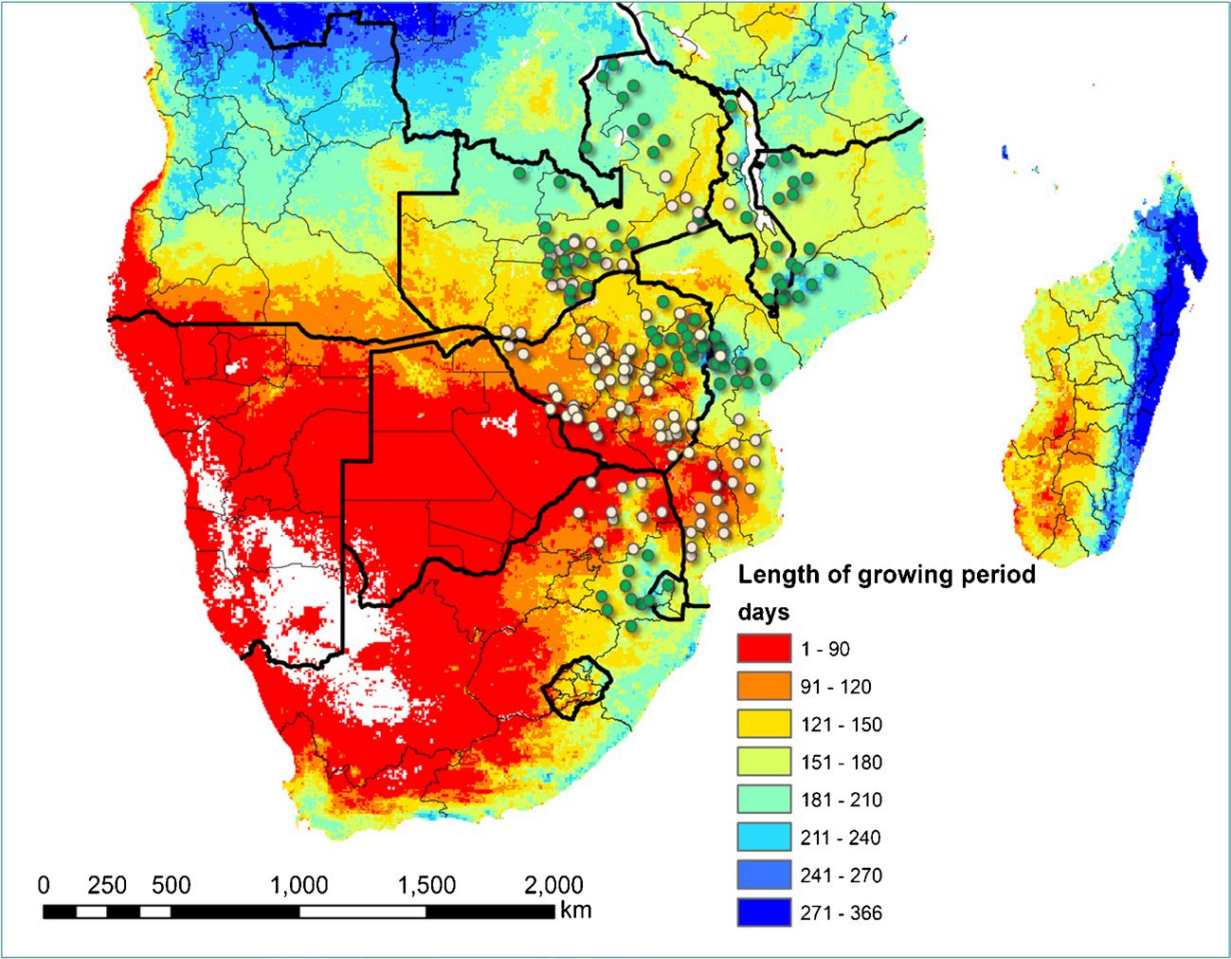
Spatial distribution of the maize on-farm testing sites in southern Africa. The green dots are for the target product profile (TPP) 1, Intermediate to late maturity. The light-yellow color is for TPP 2—early maturity.

Overall, the least-significant difference (LSD) was about 20% of the trials’ mean and the broad-sense heritability was above 0.6 for grain yield. These results indicate a significant improvement in terms of data quality compared to previous on-farm trials.

The network expansion also enabled the farmers’ sampling strategy to be improved, leading to a higher representativeness of women, as well as prevailing crop management practices (Voss et al. 2023). Social scientists in the delivery teams noted that tricot reduces issues around gender and age norms compared to the group-based PVS approach used in the past. The so-called “leadership effects” prevent younger persons and women to contradict or challenge the views of men or senior persons in a group (Richards 2005). As tricot involves farmers on an individual basis, this effect is attenuated, allowing women and younger persons to be more outspoken about their experience with their tricot plot. To ensure inclusiveness, it has been important to limit plot size, so that farmers with small landholdings can also participate. For small grain crops there has been a tendency to propose on-farm trial protocols with large plots (to address the impact of pests), which could potentially exclude women from participation, in spite of their import role in growing these crops.

The second case illustrates the experience of the cowpea (*Vigna unguiculata* (L.) Walp.) breeding network led by IITA in West Africa. In cowpea, farmer inputs are required at two critical phases: (i) TPP design, which requires farmers’ feedback, allowing breeders to capture farmer-preferred traits at the very start of variety development; and (ii) testing phase, which demands farmers’ active participation in the variety selection process. Previously, cowpea PVS generated inadequate information to feed these two important breeding phases. Like in the maize example, PVS was often established as small demonstration plots in selected central locations where farmers from sampled villages would manage the trials and rate the varieties’ performance (Ishikawa et al. 2019). In 2021, the cowpea breeding program adopted the tricot approach. The breeding program was able to evaluate 18 candidate varieties in a tricot trial involving 320 farmers from 16 local government areas in three northern states in Nigeria. Key performing varieties were identified, one of the varieties (IT13K-1308-5) was released in 2022 as “SAMPEAE 21” in Nigeria using tricot data as evidence for on-farm performance.

In the third case, tricot has been adopted by One Acre Fund as a mechanism for accelerating variety recommendations for their clients. One Acre Fund is a social enterprise dedicated to serving farmers in East Africa, with 1.5M clients across nine countries. Since 2019, One Acre Fund has deployed more than 30 tricot trials, engaging with 3289 farmers in evaluating more than 85 unique potato, maize, soybean, and bean varieties. Prior to adopting the tricot methodology, variety trials utilized randomized complete block designs (RCBD). The switch to tricot has enabled far more insight into the importance farmers and consumers assign to various traits. Furthermore, integrating tricot has been a means of accelerating the variety screening process relied upon to deliver varieties with preferred traits to their clients, due to the fact that a wider range of varieties (treatments) can be tested compared to RCBD (in which plot space is a key constraint). Since 2021, One Acre Fund has used the data generated from the tricot trials to support the scaling of new varieties for potato (Kirundo and Ndamira) and maize (APIS 610, APIS 630), as well as at least three that are recommended for scaling in Rwanda. One Acre Fund plans to update varietal research on other crops by expanding the use of tricot to sorghum and soybean in 2024. Other One Acre Fund countries are also considering adopting the tricot methodology for their variety screening.

## Challenges in mainstreaming

**Fig. 6:**
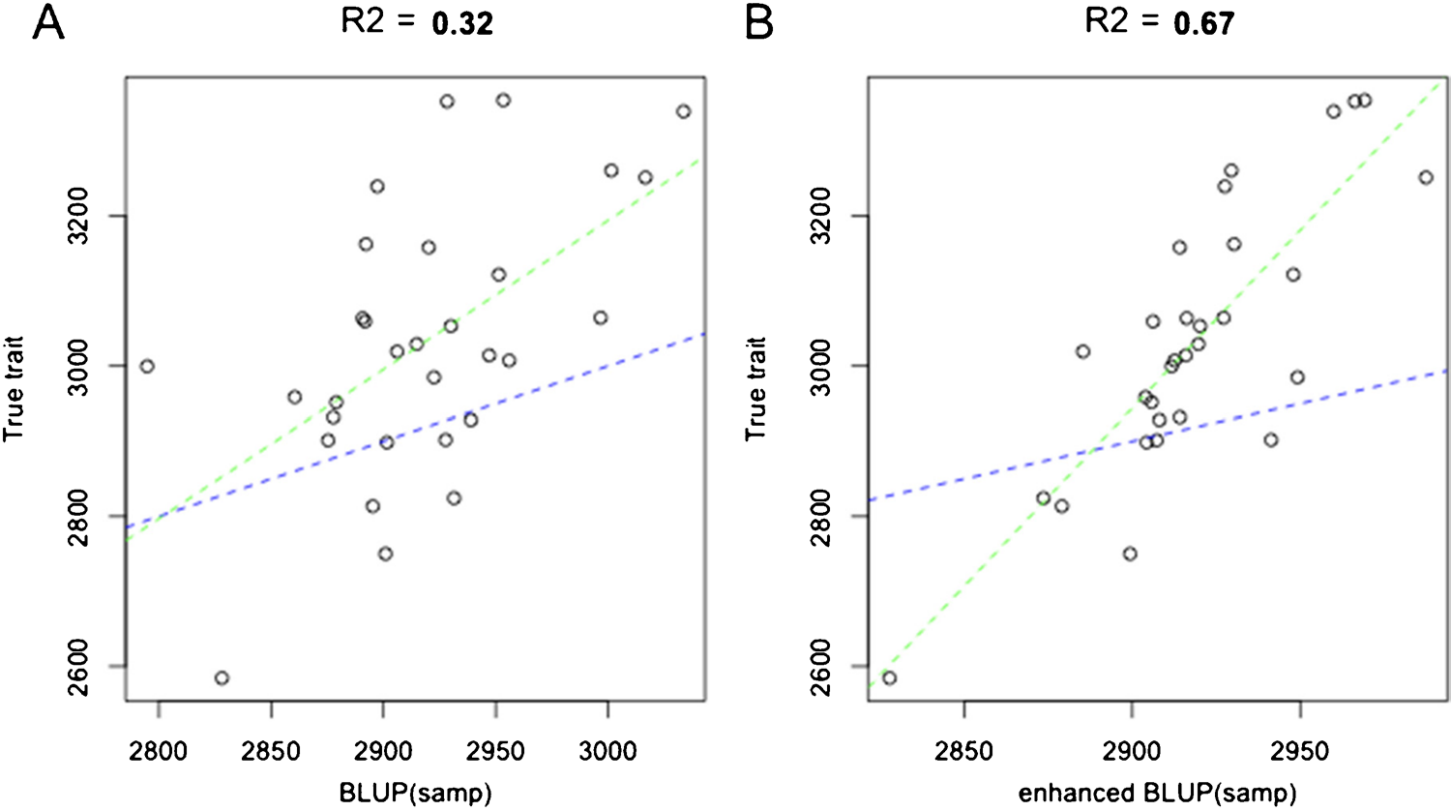
Relation between simulated grain yield and the log-worth values estimated based on ranking of yield observed in a tricot experiment. **A** Best linear unbiased estimators (BLUPs) based on 30 plot samples, and **B** “enhanced” results which are the predictions from a linear model with BLUPs as response and log(worth) as explanatory. Blue dotted line is the 1:1 intercept and the green line the regression line.

One of the challenges that the teams encountered was that breeding programs objected to relying on only farmer-generated rankings, which do not capture the performance of tested technology options on an absolute scale (especially, yield in t ⋅ ha^−1^). This makes it more difficult to translate trial results into monetary value terms. This has been particularly a discussion point with the variety release authorities in different countries in reaction to our proposition of tricot as an improved method for on-farm testing for variety release. Different approaches have been proposed to address this. Field agents can measure yields on a subset of the fields to keep the effort needed reasonable. Recently, van Heerwaarden et al. (2023) demonstrated with empirical data from groundnut tricot trials in Tanzania that ranking data and metric data can be combined. The ranking data would “lend” statistical power to the metric data collected in a subset of tricot plots (Fig. 6). Also, farmers can measure yields themselves using different methods, such as volumetric measurements (counting how many tin cans of grains each variety produces) or spring scales for bulky root and tuber crops, such as cassava. Currently, the teams are testing different approaches to collect reliable and valid farmer-led yield estimations with case studies in maize, common bean, and cassava. Preliminary results indicate a relatively good agreement between measured and farmer-led estimated yields. Mainstreaming tricot into the breeding programs helped to develop and validate on-farm trial protocols based on TPPs. Trials were designed and implemented following a protocol defined in common agreement by breeders, socio-economists, and other stakeholders. The protocols also include a suite of core traits (e.g., yield, overall appreciation, marketability), socio-economic indicators (e.g., gender decisions, management practices), and plot characterization (e.g., previous land use, slope) collected in all trials, which facilitate scaling and pooling data across trials, geographies, and crops (Brown et al. 2020, 2022).

The integration of tricot into breeding networks also supported the development of new data-driven approaches. Breeding teams required the collection of continuous on-farm data (e.g., grain yield, plant population). A useful property of the Plackett-Luce model is that the *worth* estimates, that can be efficiently obtained for each variety, provide a direct estimate of the unobserved genetic values of the trait being ranked. This can support breeding teams in estimating on-farm genetic gains, a key metric for evaluating the performance of breeding programs (Atlin et al. 2017). As can be seen from the simulated results in Fig. 6, not only does a linear relation between the true trait value and *log-worth* do exist, but the inferred differences in the latent trait scale systematically with the plot-level residual standard deviation (i.e., the observational error on the trait being ranked), which suggests that the true scale of differences may be inferred from ranking data if the level of error is known. New sub-sampling techniques are under development to support this process and generate reliable data without increasing labor and costs.

Tricot represents a divergence from traditional pathways for decision-making in agricultural technology adoption. Therefore, a high level of capacity investment and trust across implementing partners are required. Engaging extension officers in several rounds of training is important to ensure the quality of the data. It also requires institutional partners to take a more “hands-off” approach in technological terms, accepting and studying variation in on-farm practices and context rather than trying to reduce it. Also, tricot requires a balanced partnership between institutions and farmers. Results are derived directly from farmers, which can be disorienting for trial managers, who may be forced to go against ingrained perceptions of farmers’ (in)capacity to effectively manage trials. It requires a certain level of trust by partners in the approach and its outputs and recognizing that the more hands-off approach enables external validity (Kool et al. 2020). Not all breeding programs are used to do substantial on-farm testing. Several programs faced challenges in producing enough seeds from non-commercial varieties (breeding lines) in sufficient volume. Seed quality assurance is important; it needs to be routinely conducted and is an essential part of capacity building delivered to extension officers. Implementing tricot with vegetables requires extra care on logistics as these crops tend to have a faster growth rate. Data collection should focus on addressing the most important crop stages to avoid farmer burnout due to excessively exhaustive data collection.

Farmer motivation remains an important topic in discussions around implementation. Breeding programs are currently exploring the use of tricot or similar approaches in on-farm selection during early stages in the crop improvement process, as mentioned above (de Sousa et al. 2021). This also means that farmers will receive genetic materials that are potentially far from variety release, which may mean they obtain very low yields from their trial plots and the seeds are not immediately of interest. Previous studies show that farmers’ motivation to participate in tricot is mainly related to access to seeds and information (Beza et al. 2017). More research on farmer motivation is needed. Care should be taken to avoid extrinsic motivators that “crowd out” intrinsic motivators (Hennessey et al. 2015). A classic example of this is that providing monetary incentives to blood donors can reduce their willingness to provide blood, because donors are motivated by solidarity rather than money (Titmuss 1970). In the context of on-farm testing, something similar may happen when payments generate the expectation that trials are merely transactional events, rather than a space for reciprocal sharing, joint curiosity, and enjoyable professional interactions. However, there may be extrinsic motivators that “crowd in” intrinsic motivation, because they provide resources that enable even deeper engagement (Hennessey et al. 2015). The suggestion to enhance local recognition (see above) moves in this direction. Future work could be directed at identifying motivators that reliably enhance farmers’ intrinsic motivation across different contexts.

Within the CGIAR, current discussions focus on the need for a transdisciplinary breeding management system, where disciplines and institutions are attributed a clear role and decision right at each stage of the breeding process. The tricot approach can become an excellent way to socially and gender-inclusively identify stakeholder representatives to be consulted especially at the first (product profile design) and later (testing of advanced clones for variety release) breeding stages. Such crop-user representatives are currently identified as important partners to incorporate within the transdisciplinary management system that was put forward by the CGIAR Excellence in Breeding platform as a story of excellence, and is now being scaled within CGIAR-NARS-Small and Medium Enterprise (SME) breeding networks as part of the CGIAR Accelerated Breeding Initiative and supporting RTB breeding investment. Such an institutional status could further motivate the participation of tricot participants and integrate crop users more symmetrically into the breeding process as part of a farmer network that can be maintained over time.

The last important point we mention here is the need to build data science capacity, which is often still limited in NARES breeding programs. Developing analytical approaches in R enables open research and reproducibility. However, despite extensive training and the availability of code, analysis bottlenecks are still an issue and require close interaction with scientists and data analysts at partner institutions. Close interactions could enable tacit knowledge transfer in developing new analytical approaches, database management, and day-to-day issues in data science (Leonelli et al. 2017; van Etten et al. 2023). Data science needs to be more fully integrated into plant breeding curricula in the Global South to overcome current challenges. Also, it may help to make the statistical models available through interactive interfaces that do not require the ability to write analytical scripts.

## Informing product design

The utilization of tricot has also gone beyond on-farm testing to identify consumers’ preferences and market demands. Building on previous experience of on-farm consumer testing, CIP applied tricot in consumer testing trials with sweet potato genotypes in local markets (centralized) and households (decentralized) in Ghana and Uganda (Moyo et al. 2021). The results showed that preferences were different across the administrative regions of the countries studied. These findings supported the breeding teams at CIP in advancing new sweet potato varieties with superior performance from consumers’ perspectives. More recently, at IITA, Olaosebikan et al. (2023) used tricot to identify consumers’ preferences on *gari* (cassava root flour) and *eba* (cooked cassava root flour balls), both food staple in Cameroon and Nigeria. In this study, the genotypes’ biophysical features measured at the lab (e.g., color spectrum L*a*b*, cohesiveness, adhesiveness, and hardness) were linked to the tricot data collected in *eba* tasting trials at local markets in the countries of study following the approach developed by Yildiz et al. (2020). The results showed that consumers’ preferences in these countries are driven by higher cohesiveness and brightness in *eba* products especially in Cameroon, where products with lower redness and yellowness are also preferred. In Nigeria, higher *eba* hardness and springiness values were preferred. In another study in Nigeria, Alamu et al. (2023) also identified that ethnic groups and their specific practice of processing the food product had a stronger influence on *eba* preference explained with tricot data linked to *eba*’s biophysical features.

Previously, we discussed the advantages of on-farm testing in guiding breeding programs to more optimal choices and priorities based on farmers’ needs. Yet it is generally applied at the end (or late stages) of the breeding process and is limited to evaluating breeding program choices made when the TPP was designed. Rutsaert et al. (2023) adapted the tricot approach to create market intelligence research in which *product ideas* instead of *actual crop varieties* or *food products* are evaluated by farmers. Through a video-based *product concept* testing method, eight hybrid maize variety concepts were tested among 2400 farmers in Kenya and Uganda. Farmers were exposed to a set of three videos simulating an agrodealer selling a variety concepts—i.e., a description of an actual or hypothetical variety and its potential uses and benefits for farming—and had to indicate which concept they would prefer to adopt or not adopt in their farm if it were available. This approach allowed us to explore alternative breeding priorities versus the ones that are currently identified for maize, i.e., white maize targeted at food with high pest and disease tolerance. For example, concepts were designed around dual-purpose maize for food fodder or maize ideal for chicken feed. Other concepts were designed around different production systems, describing a variety tailored to intercropping or an early-maturing variety to avoid drought instead of being resistant to it. Country and gender characteristics influenced these farmers’ choices, indicating the ability of tricot data and the analytical approach to recognize socio-economic heterogeneity to support decision-making in agricultural product management.

## Gender and socio-economic heterogeneity

Another work stream has focused on gender and socio-economic heterogeneity in tricot trials. Social heterogeneity is important in on-farm testing, because it can lead to divergent crop variety needs and preferences for different user groups. Such differences can be due to social factors influencing different system levels: crop management, cropping systems, farming systems, and livelihood systems. For example, a survey in Zimbabwe found that women tend to have a stronger preference for short-maturity maize varieties than men and are more interested in intercropping maize with other crops (Cairns et al. 2022). Previous studies had tried to study gender-based differences by inviting farmers to observe researcher-managed variety trials, but had not been able to elucidate relevant gender-differentiated differences. Tricot trials could be instrumental in generating such insights, in contrast with more conventional PVS, particularly before varieties are released, thereby complementing survey-based studies. This means that (i) trials should be carried out with farmers (and other stakeholders) who represent different crop-user segments; and (ii) the trials should be accompanied by socio-economic data collection and analysis.

The work on cassava in Nigeria has been especially important in improving sampling strategies and has been conducted alongside research to characterize cassava users’ trait preferences (Teeken et al. 2021b; Balogun et al. 2022) and food product preferences (Teeken et al. 2018, 2021a, b; Balogun et al. 2022). A combination of interviews with community leaders, snowball sampling, and focus group discussions was used to compile lists of candidate participants representing different user segments, taking into account their level of experience and expertise and locally distinguished social groups as informed by key informant interviews with village leads. These lists were then sampled randomly, proportionally balancing across these user segments. Given the gendered tasks especially related to processing (Teeken et al. 2021a), the post-harvest processing evaluation of the tricot trials in Nigeria and the subsequent food product evaluations needs to be done by the person who normally carries out that work rather than working only with the identified individual tricot participant. This practice ensures an assessment based on expertise and gendered working conditions, from planting up to food product quality evaluation. Furthermore, farmers in Nigeria have proposed planting their own preferred plot next to the three varieties in the tricot trial as they stated that it would allow them to better assess each of the three clones in relation to their local variety. This suggestion has been adopted in Nigeria. Similar protocols used for tricot and other types of cassava-user research in Nigeria need to be developed for other tricot trials. This research is already leading to a much more detailed understanding of social drivers of crop variety preferences.

Another important aspect of this research has been to adopt a standardized set of socio-economic questions, derived from a light version of the Rural Household Multiple Indicator Survey (RHoMIS) (Hammond et al. 2017; Teeken et al. 2021b). Geospatial factors should also be considered to ensure geographic representativeness, avoiding the exclusion of farms in more marginal or better-equipped areas in terms of road access or agricultural potential. The more diverse the farms are across multiple dimensions of variation, the higher the potential of generating a deeper understanding of user preferences and their underlying potential drivers. At the same time, practical considerations (access to communities, logistics) and transport costs, especially for vegetatively propagated crops with bulky and perishable planting materials, need to be well considered. Ongoing work is focused on bringing the different sampling aspects together in a stepwise, digitally supported sampling strategy.

## Conclusions

Through a systematic scaling approach, tricot has gone from a piloting exercise for participatory variety selection in India and Ethiopia to the leading on-farm testing methodology in the NARES-CGIAR network. One important lesson we draw from this experience is that creating routines is crucial for scaling. An alternative approach is to adopt a principle-guided open-ended focused approach to local operationalization (Richardson et al. 2022), which can be expected to be better, because it can be reshaped to fit particular goals and contexts in intensive consultation and negotiation with different stakeholders. In our experience and context, such an approach requires high levels of interdisciplinary expertise and it is vulnerable to obstacles and broken phone effects along the different steps in the long chain of on-farm testing, from experimental design to decision-making. These obstacles are often exacerbated by staff turnover. Some degree of standardization makes it possible to overcome several of these obstacles by supporting the process with digital media, easing trial coordination, experimental design, data management, and data analysis. Therefore, our approach has been to streamline good practices and tools through cumulative learning into a coherent, robust approach that works in the majority of contexts, allowing for flexibility at key points. This approach has enabled a large scaling effort that should lead to improved decision-making in crop improvement and increased use of improved varieties in the coming decades.

Key research and innovation issues that need to be addressed in future work are (i) how to continuously improve alignment between concepts and motivation between the scientific, technical, and farmer communities; (ii) how to ensure sustainability in partnerships, institutionalization, and financing of on-farm testing; (iii) how to manage the balance between hands-off requirements for external validity and farmer management, on the one hand, and accountability for trial success and concurrent improvements in crop management, on the other; (iv) how to use tricot to study interactions between crop genotypes, environment, and crop management (GxExM), especially for climate adaptation and risk management; (v) how to iteratively improve the inclusion of tricot participants to become more representative of gender and other socio-economic diversity and the target population of environments in a cost-efficient way; and (vi) how breeding programs respond to the on-farm testing results and adjust breeding efforts to better serve farmer needs. Promoting tricot participants to stakeholders with roles and decision-making rights at different stages of the breeding process has been highlighted as an institutional innovation that could close the status and institutional gap between breeders and crop users.

Over the last 12 years, the tricot approach has been tested in different contexts, demonstrating its ability to provide reliable data at scale. An important benefit of tricot is the possible reduction of trial costs, a key bottleneck in institutional scaling of on-farm testing. Cost analyzes in Rwanda already demonstrate a cost reduction of up to 27% due to tricot. Further cost reductions are possible if farmer networks are maintained over time, if they are serviced through channels that are also used for other means (e.g., credit provision, access to markets), to be facilitated by building win-win partnerships including NGOs and the private sector, and if they can reach economies of scale and scope by testing varieties and other options for multiple crops. Tricot would make it possible for breeders and agronomists to “outsource” trials to farmer-facing organizations. Alternative business models have already been introduced in the US context by organizations such as the Farmer Business Network, FIRST (Farmers’ Independent Research of Seed Technologies) and SeedLinked. The latter uses an approach inspired by tricot for its trials. The CGIAR is exploring alternative business models following this trend focusing on the Global South. Private extension organizations are also applying and scaling tricot across multiple crops and countries in Africa. Tricot provides a framework that can make breeding more demand driven and inclusive of different crop users (Donovan et al. 2022; Polar et al. 2022), thus maximizing the contribution of plant breeding to social, gender, nutritional, economic, climate change, and environmental impact areas.

## Data Availability

Data used to perform the analysis for this paper are available from Zenodo (de Sousa 2022).

## Code availability

Code used to perform the analysis for this paper is available from Zenodo (de Sousa 2022).
